# Avian influenza virus exhibits distinct evolutionary dynamics in wild birds and poultry

**DOI:** 10.1186/s12862-015-0410-5

**Published:** 2015-06-26

**Authors:** Mathieu Fourment, Edward C Holmes

**Affiliations:** Marie Bashir Institute for Infectious Diseases and Biosecurity, Charles Perkins Centre, School of Biological Sciences and Sydney Medical School, The University of Sydney, Sydney, NSW 2006 Australia

## Abstract

**Background:**

Wild birds are the major reservoir hosts for influenza A viruses, occasionally transmitting to other species such as domesticated poultry. Despite an abundance of genomic data from avian influenza virus (AIV), little is known about whether AIV evolves differently in wild birds and poultry, although this is critical to revealing the dynamics and time-scale of viral evolution. In particular, because environmental (water-borne) transmission is more common in wild birds, which may reduce the number of replications per unit time, it is possible that evolutionary rates are systematically lower in wild birds than in poultry.

**Results:**

We estimated rates of nucleotide substitution in two AIV subtypes that are strongly associated with infections in wild birds – H4 and H6 – and compared these to rates in the H5N1 subtype that has circulated in poultry for almost two decades. Our analyses of three internal genes confirm that H4 and H6 viruses are evolving significantly more slowly than H5N1 viruses, suggesting that evolutionary rates of AIV are reduced in wild birds. This result was verified by the analysis of a poultry-associated H6 lineage that exhibited a markedly higher substitution rate than those H6 viruses circulating in wild birds. Interestingly, we also observed a significant difference in evolutionary rate between H4 and H6, despite frequent reassortment rate among them.

**Conclusions:**

AIV experiences markedly different evolutionary dynamics between wild birds and poultry. These results suggest that rate heterogeneity among viral subtypes and ecological groupings should be taken into account when estimating evolutionary rates and divergence times.

**Electronic supplementary material:**

The online version of this article (doi:10.1186/s12862-015-0410-5) contains supplementary material, which is available to authorized users.

## Background

Influenza A viruses infect a wide range of animals including humans, pigs, horses, and poultry. However, with the exception of a small number of viruses recently described in bats [1,2], wild (water) birds are the natural reservoir for all haemaggluttinin (H) and neuraminidase (N) subtypes, harbouring 16 of the former and 9 of the latter [3-5]. With approximately 10,000 bird species present in terrestrial and aquatic environments, the often large sizes and densities of bird populations are a boon for the spread of infectious diseases such as influenza. Avian influenza (AIV) is believed to be largely asymptomatic in wild birds such as waterfowl, causing mild or no symptoms, such that most AIVs in these species can be considered to be low pathogenic avian influenza (LPAI). In contrast, poultry (chicken and turkey) are not considered as natural hosts for AIV, but can be infected with both LPAI and high pathogenic (HPAI) strains, notably H5 and H7. HPAI outbreaks may result in high mortality through severe respiratory distress, with major economic costs to the agricultural industries. Strikingly, despite the diversity of H and N types in wild birds, only a small number – H1, H2, H3, N1 and N2 – have evolved sustained transmission in humans, indicating that there are major adaptive barriers associated with the bird to mammal species jump [6].

It is similarly noteworthy that AIV outbreaks in poultry have only been associated with a relatively small number of H and N subtypes, suggesting that there are important species barriers within birds. A question that has been less frequently addressed is whether evolutionary patterns and processes also differ between wild birds and poultry? Indeed, there are a number of factors that could create important evolutionary differences between AIV in wild birds and poultry, notably reflecting differences in the mode of transmission and population structure. In particular, the transmission of AIV between wild birds largely occurs through an indirect faecal-oral route, in which, following excretion, the virus sits in an aquatic environment before infecting another host [2,7,8]. Importantly, there is growing evidence that AIV is able to remain in these water environments for several weeks, although this is dependent on the physico-chemical characteristics of the water source in question, and the presence of microorganisms which limit AIV infectiousness [7-11]. Critically, while present in the environment, AIV will effectively be in a “latent” state, such that there will be no viral replication and no mutational accumulation. In contrast, AIV transmission in poultry is more likely to be a function of close contact (including airborne) and rapid faecal-oral transmission, without an extended latent period in water. Hence, a simple prediction from these differing modes of transmission is that evolutionary rates in AIV will be lower in wild birds than in poultry. Similarly, it is possible that the rapidity of AIV outbreaks in poultry increases the number of viral replications per unit time which, depending on the fitness of the mutations involved, may also increase evolutionary rates. Finally, selection pressures may also differ between wild birds and poultry. High-density poultry populations are dominated by the presence of a single viral subtype (e.g. H5, N7) within which there may be strong selection pressure on the virus to escape from increasing host immunity [12,13]. Indeed, HP H5N1 has been characterized by steady antigenic change (drift) since its emergence [14]. In contrast, weaker antigenic drift might be expected in wild bird populations that experience concurrent infections with multiple antigenically distinct subtypes [13]. To date, however, these fundamental differences between wild birds and poultry have not been incorporated into evolutionary models, including molecular clock dating.

Most estimates of nucleotide substitution rates for influenza virus inferred using temporally sampled viruses point to rates in the order of 10^−3^ substitution per site, per year (subs/site/year), in which genes coding for the external H and N proteins have higher evolutionary rates than internal genes [15-19]. The estimation of evolutionary rates in a statistical framework relies on the applicability of a molecular clock where the substitution rate can be constrained to be constant over time, or vary across time and among lineages. Methods that model rate heterogeneity include auto-correlation of rates among lineages [20], and uncorrelated models that allow rates assigned to lineages to be drawn from an underlying probability distribution [21]. Yoder and Yang (2000) modelled rate heterogeneity among lineage (heterotachy) using local clock models, a method that was recently reinvestigated using modern Bayesian-based sampling methods [22]. This clock model allows phylogenetically related lineages to evolve at exactly the same rate. All these models are evidently oversimplifications of the substitution process and tend to provide different estimates, illustrating the need for the development of accurate model selection methods. Recently, Worobey et al. [18] used host-specific local clocks to infer the history of influenza A viruses and showed that the substitution rate of avian viruses were significantly higher than the human rate, although slightly lower or equal to the rate in swine. In this study, horses, birds, swine and humans are assumed to evolve at different rates, while viruses within each host are constrained to evolve at the same constant rate (i.e. a strict molecular clock). Critically, therefore, it was assumed that all AIVs evolve at the same rate, although the major ecological differences between wild birds and poultry noted above mean that this may be a simplification. Indeed, it is expected that the estimated substitution rates will be an average of multiple substitution rates that reflect the proportion of each viral subtype in the data set analysed.

To investigate the evolutionary rate heterogeneity of AIV, and particularly whether there is a systematic difference in rates between wild birds and poultry, we compiled data sets for the PB2, PB1, and PA internal genes representing AIV in these very different ecological contexts. These genes should be suitable for inferring substitution rates as they are the longest in the viral genome, are considered to be under moderate (predominantly negative) selection pressures, and different lineages often circulate in wild birds and poultry, facilitating the direct comparison of AIV in these two types of host species. Accordingly, for poultry, we examined sequences from the H5N1 (HPAI) subtype that has caused major outbreaks since 1997, while for wild birds we examined the commonly observed H4 and H6 subtypes (in combination with any other N type).

## Results and discussion

**Fig. 1:**
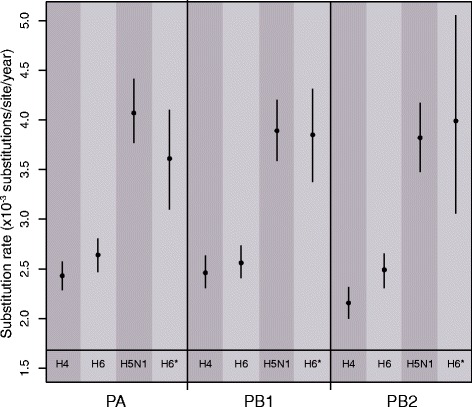
Evolutionary rates of avian influenza virus (AIV) among poultry and wild birds for three internal genes (PA, PB1, PB2). The nucleotide substitution rate per site of each gene was analysed using BEAST. Mean substitution rates and 95 % Bayesian confidence intervals estimated under strict (triangles) and lognormal (circles) molecular clocks are reported. * denotes a lineage of H6 viruses from poultry

The MCMC-based (BEAST) analysis shows that the mean nucleotide substitution rate of AIV varies between 1.87 × 10^−3^ and 4.2 × 10^−3^ subs/site/year depending on the model used. These results consistently show significant lower rates (i.e. with non-overlapping confidence intervals) in those subtypes associated with wild birds (H4, H6) compared to poultry (H5N1) (Fig. 1). To further test this separation we analysed a monophyletic group of H6 viruses (denoted H6*) that persisted in the poultry population rather than in their usual wild bird hosts. Strikingly, estimates of substitution rates in these poultry H6 viruses are similar to those in H5N1 from poultry and significantly higher than those of H6 from wild birds (Fig. 1), confirming that AIV evolutionary rates are elevated in poultry. Similarly, estimates of the mean ω (a measure of overall selection pressure) and the number of positively selected sites revealed consistent differences in selection pressures between H5N1 and the other subtypes (Table 1). In particular, the mean ω of the H5N1 genes is substantially higher than those of H4 and H6, while no significant difference was observed between PA, PB1 and PB2.

**Table 1:** Selection analyses of AIV among poultry and wild birds for three internal genes (PA, PB1, PB2)

	Mean ω			Positively selected			Ratio ω_e_/ω_i_		
	PB2	PB1	PA	PB2	PB1	PA	PB2	PB1	PA
H4	0.3	0.28	0.36	0	0	0	3.17	3.21	3.31
H6	0.27	0.36	0.29	1	0	0	3.57	2.81	2.69
H5N1	0.48	0.55	0.73	0	1	5	1.53	2.24	1.84
H6*	1.32	1.15	1.31	0	0	0	2.71	3.24	2.98

H4 and H6 data sets contain wild birds only, while H5N1 and H6* are poultry-only data sets. Mean *ω* and the number of positively selected sites for each gene of each subtype were estimated using FUBAR. Separate ratios of nonsynonymous to synonymous substitution rates for external (*ω*_*e*_) and internal (*ω*_*i*_) branches were estimated using HyPhy

This important discrepancy in evolutionary rates between those AIV subtypes that circulate in wild birds compared to poultry can be explained in at least two ways. First, this variation in rate could reflect underlying ecological differences. In particular, substitution rates may be reduced in wild birds because of the latent period associated with environmental (water-borne) transmission. In contrast, more frequent contact transmission in poultry may lead to more replications, and hence a greater number of mutations, per unit time [23]. Selection pressures may also differ between wild birds and poultry, as revealed here (Table 1), and there is evidence for host-driven antigenic drift in H5N1 [12,13] which would be expected to increase evolutionary rates, especially at nonsynonymous sites. Second, because of heightened scientific attention, H5N1 has been more densely sampled than the other subtypes. It is possible that such intensive sampling may have increased the number of recently diverged sequences and hence the relative proportion of slightly deleterious mutations that are yet to be removed by purifying selection, in turn increasing rates toward the present [24]. Indeed, our analysis of selection pressures revealed that the mean values of *ω* were higher for H5N1 than for H4 and H6 (Table 1). To determine whether this increase of *ω* in H5N1 was indeed due to a higher frequency of transient deleterious mutations, or stronger positive selection, we examined *ω* values on internal and external branches of the phylogenies (Table 1). Notably, in every data set there was a significantly higher *ω* on external (*ω*_*e*_) branches compared to internal (*ω*_*i*_) branches (p-value < < 0.05). Importantly, however, the ω_e_/ω_i_ ratio of the H5N1 data sets were consistently the lowest, such that this subtype has experienced proportionally more nonsynonymous mutations on internal branches and compatible with stronger positive selection. Hence, the elevated substitution rates in H5N1 are unlikely to be a function of sampling density.

Our analysis further reveals that the mean substitution rate of the H6 subtype is consistently higher than that observed in H4 subtype. This disparity is surprising considering that both subtypes infect the same wild bird species and continuously reassort such that they often do not form distinct monophyletic groups, and have nearly identical selection profiles. Interestingly, however, chickens may be infected more frequently by the H6 than the H4 subtype. Taking the PA gene as an example, 7 % of all H6 sequences available on GenBank come from chickens, while the corresponding number for H4 is 0.3 % (although all poultry sequences were excluded from this analysis). While the more frequent circulation of the H6 lineage in the poultry population could explain the elevated rate in this subtype, this clearly needs to be confirmed on a larger and less biased data sample.

Also of note was that estimates of the mean substitution rate under an uncorrelated lognormal clock model are significantly higher than those estimated under a strict clock model. Considering the size of the data sets, the calculation of Bayes factors with computer intensive methods such as path sampling [25] is prohibitive. Instead we computed the AICM for each model (Additional files 1: Table S1, S2, and S3) and the results reveal the presence of rate heterogeneity in most data sets.

It is not straightforward to compare our estimates of the times to common ancestry to those of Worobey et al. [18] since the most recent common ancestor of all avian viruses (as analysed there) is not necessarily the MRCA of the subtypes analysed here. For example, H5N1 is the most recent lineage and originated between 12 and 16 years ago depending on the molecular clock model used, while the H4 and H6 subtypes have a tMRCA of at least 50 years but do not predate 1900 as suggested by the previous local clock analysis (Table 2). However, it is apparent that our estimates of root age are consistently younger than those of Worobey et al., suggesting that accounting for rate heterogeneity can lead to surprisingly different results [26].

**Table 2:** Estimates of time to the most recent common under the best model for each AIV data set

Segment	Subtype	Population	Clock	Mean	Lower	Upper
PA	H5N1	Skyride	Lognormal	1999	1998	2000
	H4	Skyride	Lognormal	1962	1960	1964
	H6 wild birds	Skyride	Lognormal	1961	1959	1964
	H6 poultry	Skyride	Strict	1994	1993	1995
PB1	H5N1	Skyride	Lognormal	2000	2000	2001
	H4	Skyride	Lognormal	1963	1961	1965
	H6 wild birds	Constant	Lognormal	1942	1931	1951
	H6 poultry	Constant	Strict	1994	1993	1995
PB2	H5N1	Skyride	Lognormal	2000	2000	2001
	H4	Constant	Lognormal	1905	1874	1934
	H6 wild birds	Constant	Lognormal	1909	1876	1934
	H6 poultry	Constant	Lognormal	1993	1988	1998

Mean and 95 % Bayesian confidence intervals are reported for each gene. The population size and clock prior are reported

## Conclusions

The estimates of nucleotide substitution rate presented here reveal a more complex picture of the evolutionary processes driving the evolution of avian influenza virus than previously thought. Most notably, it is clear that a single substitution rate cannot be applied to wild birds and poultry equally, as the evolutionary rates are consistently lower in the former and which may reflect a greater role for environmental transmission. Hence, it is evidently incorrect to impose a single substitution to AIV sampled from hosts as diverse as wild birds and poultry, and that molecular clock estimates based on a single rate may be erroneous. Similarly, rates differ significantly between the strict and relaxed clock models. It is therefore likely that the evolutionary dynamics of avian influenza virus can only be captured by new models that allow rates to vary in a more complex manner along lineages and which reflect changes in the underlying ecology.

## Methods

### Data

To determine whether there are significant differences in evolutionary rates between AIV from wild birds and poultry we focused on internal rather than external (i.e. H, N) proteins because the former are longer and less subject to positive selection that will likely have a major impact on evolutionary dynamics. Accordingly, full-length nucleotide sequences encoding the PB2, PB1, and PA proteins of AIV were obtained from the Influenza Virus Resource database at NCBI (GenBank) [27]. Four separate data sets were created for each gene based on their subtypes (H5N1, H4, and H6) and hosts (wild birds versus poultry). The H5N1 data sets were constructed using sequences isolated exclusively from poultry (i.e. chicken and turkey), while in the H4 and H6 data sets the small numbers of chicken and turkey (i.e. poultry) sequences were removed to obtain data sets composed exclusively of wild bird samples. An additional data set was created using H6 sequences isolated from chickens that formed a distinct monophyletic group, and hence which could be used to independently assess whether evolutionary rates are elevated in poultry. Multiple sequence alignments were generated using MAFFT version 7 [28] and manually edited using Seqotron [29], with final alignment lengths of 2151 bp, 2271 bp and 2280 bp for PA, PB1 and PB2, respectively. Total data set sizes were: H5N1: PA = 479 sequences, PB1 = 411, PB2 = 391; H4: PA = 811, PB1 = 840, PB2 = 797; H6 (wild bird only): PA = 557, PB1 = 550, PB2 = 545; H6 (poultry only): PA = 36, PB1 = 35, PB2 = 32. For each gene, H4, H6, and H5N1 final data sets were combined and the phylogenetic history was investigated using the neighbor joining method. The tree files are available as supplementary files (Additional files 2, 3, and 4). Finally, for each gene, we provide neighbor-joining trees containing every H4, H6, and H5N1 sequence available on GenBank, and which shows the distribution of poultry versus wild bird viruses among subtypes (Additional files 5, 6, and 7).

### Phylogenetic analyses

Phylogenetic trees were inferred using maximum likelihood available in Physher [26] employing the generalised time-reversible (GTR) substitution model and a discretised gamma distribution (4 categories) of rates across sites (Γ_4_). Using the year of isolation as sampling time, temporal outliers possibly indicative of sequencing errors were removed by inspecting the correlation coefficient between regressions of root-to-tip divergence and sampling times using Path-O-Gen [30]. Correlation coefficients ranged between 0.66 and 0.9 in all cases.

Estimates of nucleotide substitution rate were performed using the Markov chain Monte Carlo (MCMC) method available in BEAST [31] with the BEAGLE library [32]. All analyses were performed under the GTR+ Γ_4_ model of nucleotide substitution, with the exception of the H6 poultry data sets where the simpler HKY+ Γ_4_ model was used due to convergence issues. Every data set were analysed using the non-parametric Bayesian skyride or constant population size prior [33], and either a strict or uncorrelated lognormal relaxed clock [21]. An exponential prior was placed on the substitution rate with mean 3 × 10^−3^ subs/site/year. For each data set at least two chains of 100 million generations were run and an appropriate number of samples were discarded as burn-in after visually assessing the convergence of chains using Tracer version 1.6 [34]. Finally, independent runs were combined using LogCombiner. Means and 95 % highest posterior density (HPD) intervals of the substitution rate and divergence time parameters were calculated from the posterior sample. We compared models using the approximate AICM [25] implemented in Tracer.

To evaluate the strength of natural selection across sites, we used the fast unconstrained Bayesian approximation (FUBAR) method [35]. The Muse-Gaut (MG4) codon model of substitution [36] incorporating general nucleotide substitution biases (MG94 × GTR) and the phylogeny estimated from the nucleotide data was used to investigate site-specific selection. The sign of selection acting on the sequences was assessed using the ratio *ω* of the relative rates of nonsynonymous (*β*) to synonymous (*α*) nucleotide substitutions per site. In addition, we analysed differences in selective pressures between internal and external lineages (i.e. any branch leading to a taxon) using a two-ratio model implemented in HyPhy [37], and where an elevated *ω* on internal versus external branches is indicative of stronger adaptive evolution. The difference in goodness-of-fit between the two-ratio and one-ratio model (i.e. where this is a single *ω* for all branches) was evaluated using the likelihood ratio test with one degree of freedom.

## Availability of supporting data

The data sets supporting the results of this article are available in the Zenodo repository, http://dx.doi.org/10.5281/zenodo.18622.

## Additional files

Additional file 1:
**Supplementary Table 1 to 3.**
Additional file 2:
**Neighbour-joining tree of the PA gene segment data set.** Neighbour-joining tree in newick format containing the H4, H6, and H5N1 viruses analysed in this study. This can be opened with FigTree (free at: http://tree.bio.ed.ac.uk/software/figtree/). Taxa are colour-coded by subtype (H5N1: red, H4: blue, H6 wild bird: black, H6 poultry: green). Additional file 3:
**Neighbour-joining tree of the PB1 gene segment data set.** Neighbour-joining tree in newick format containing the H4, H6, and H5N1 viruses analysed in this study. This can be opened with FigTree (free at: http://tree.bio.ed.ac.uk/software/figtree/). Taxa are colour-coded by subtype (H5N1: red, H4: blue, H6 wild bird: black, H6 poultry: green). Additional file 4:
**Neighbour-joining tree of the PB2 gene segment data set.** Neighbour-joining tree in newick format containing the H4, H6, and H5N1 viruses analysed in this study. This can be opened with FigTree (free at: http://tree.bio.ed.ac.uk/software/figtree/). Taxa are colour-coded by subtype (H5N1: red, H4: blue, H6 wild bird: black, H6 poultry: green). Additional file 5:
**Neighbour-joining tree of the PA gene segment complete data set.** Neighbour-joining tree in newick format containing the H4, H6, and H5N1 viruses analysed in this study. This file can be opened with FigTree (free at: http://tree.bio.ed.ac.uk/software/figtree/). Taxa are colour-coded by subtype (H5N1: red, H4: blue, H6: black). Additional file 6:
**Neighbour-joining tree of the PB1 gene segment complete data set.** Neighbour-joining tree in newick format containing the H4, H6, and H5N1 viruses analysed in this study. This file can be opened with FigTree (free at: http://tree.bio.ed.ac.uk/software/figtree/). Taxa are colour-coded by subtype (H5N1: red, H4: blue, H6: black). Additional file 7:
**Neighbour-joining tree of the PB2 gene segment complete data set.** Neighbour-joining tree in newick format containing the H4, H6, and H5N1 viruses analysed in this study. This file should be opened with FigTree (free at: http://tree.bio.ed.ac.uk/software/figtree/). Taxa are colour-coded by subtype (H5N1: red, H4: blue, H6: black).
