# Supplementary material for: Avian influenza virus exhibits distinct evolutionary dynamics in wild birds and poultry
Source: BMC Evol Biol. 2015 Jun 26;15:120. doi: 10.1186/s12862-015-0410-5 (PMC4481119; doi:10.1186/s12862-015-0410-5)
Supplement: Additional file 1: — Supplementary Table 1 to 3. [file 12862_2015_410_MOESM1_ESM.docx]

Table 1: Estimates of substitution rate and time to the most recent common under different population size and clock priors for segment PA.

Mean and 95% Bayesian confidence intervals (between brackets) are reported for each gene. The best model for each data set according to the AICM is highlighted.

| Subtype | Population | Clock | Rate (95% HPD) | Age (95% HPD) | AICM | SE |
| --- | --- | --- | --- | --- | --- | --- |
| **H5N1** | **GMRF** | **Lognormal** | **4.07 (3.77-4.41)** | **1999 (1998-2000)** | **60499.44** | **15.97** |
| H5N1 | GMRF | Strict | 3.55 (3.29-3.81) | 1994 (1993-1995) | 61082.31 | 11.52 |
| H5N1 | Constant | Lognormal | 3.75 (3.4-4.08) | 1988 (1981-1993) | 60533.2 | 16.05 |
| H5N1 | Constant | Strict | 3.51 (3.27-3.78) | 1990 (1989-1992) | 61008.66 | 10.32 |
| **H4** | **GMRF** | **Lognormal** | **2.43 (2.29-2.57)** | **1962 (1960-1964)** | **130465.84** | **18.5** |
| H4 | GMRF | Strict | 2.02 (1.92-2.12) | 1939 (1935-1942) | 130968.8 | 18.76 |
| H4 | Constant | Lognormal | 2.26 (2.11-2.4) | 1912 (1887-1936) | 130587.7 | 18.77 |
| H4 | Constant | Strict | 1.99 (1.9-2.09) | 1910 (1902-1918) | 130762.85 | 16.50 |
| **H6** | **GMRF** | **Lognormal** | **2.64 (2.47-2.8)** | **1961 (1959-1964)** | **116791.06** | **18.70** |
| H6 | GMRF | Strict | 2.18 (2.08-2.29) | 1938 (1934-1941) | 117358.94 | 12.05 |
| H6 | Constant | Lognormal | 2.37 (2.21-2.54) | 1919 (1896-1939) | 116812.19 | 17.22 |
| H6 | Constant | Strict | 2.12 (2.02-2.23) | 1921 (1915-1927) | 117254.95 | 11.29 |
| H6 poultry | GMRF | Lognormal | 3.72 (3.1-4.36) | 1995 (1993-1996) | 14058.32 | 1.36 |
| **H6 poultry** | **GMRF** | **Strict** | **3.61 (3.1-4.1)** | **1994 (1993-1995)** | **14039.98** | **0.82** |
| H6 poultry | Constant | Lognormal | 3.62 (2.99-4.25) | 1994 (1991-1996) | 14061.68 | 1.36 |
| H6 poultry | Constant | Strict | 3.6 (3.08-4.1) | 1994 (1993-1995) | 14045.16 | 0.87 |

**Table 2: Estimates of substitution rate and time to the most recent common under different population size and clock priors for segment PB1.**

Mean and 95% Bayesian confidence intervals (between brackets) are reported for each gene. The best model for each data set according to the AICM is highlighted.

| Subtype | Population | Clock | Rate (95% HPD) | Age (95% HPD) | AICM | SE |
| --- | --- | --- | --- | --- | --- | --- |
| **H5N1** | **GMRF** | **Lognormal** | **3.89 (3.59-4.2)** | **2000 (2000-2001)** | **55202.96** | **14.95** |
| H5N1 | GMRF | Strict | 3.57 (3.31-3.82) | 1996 (1995-1997) | 55778.91 | 12.64 |
| H5N1 | Constant | Lognormal | 3.68 (3.29-4.1) | 1987 (1971-1999) | 55264.1 | 15.33 |
| H5N1 | Constant | Strict | 3.41 (3.17-3.66) | 1988 (1985-1990) | 55556.92 | 9.44 |
| **H4** | **GMRF** | **Lognormal** | **2.46 (2.31-2.63)** | **1963 (1961-1965)** | **137349.75** | **27.86** |
| H4 | GMRF | Strict | 2.04 (1.95-2.14) | 1933 (1929-1937) | 138151.06 | 26.16 |
| H4 | Constant | Lognormal | 2.72 (2.1-2.45) | 1888 (1849-1924) | 138672.39 | 18.3 |
| H4 | Constant | Strict | 2 (1.9-2.1) | 1889 (1877-1880) | 139024.33 | 14.43 |
| H6 | GMRF | Lognormal | 2.77 (2.57-2.95) | 1963 (1961-1965) | 118487.63 | 19.16 |
| H6 | GMRF | Strict | 2.34 (2.21-2.46) | 1951 (1949-1953) | 118847.7 | 14.1 |
| **H6** | **Constant** | **Lognormal** | **2.56 (2.41-2.73)** | **1942 (1931-1951)** | **118441.82** | **17.08** |
| H6 | Constant | Strict | 2.27 (2.15-2.39) | 1942 (1938-1945) | 118722.98 | 11.55 |
| H6 poultry | GMRF | Lognormal | 3.64 (3.08-4.24) | 1994 (1991-1996) | 15718.3 | 1.533 |
| H6 poultry | GMRF | Strict | 3.83 (3.36-4.27) | 1994 (1993-1995) | 15704.12 | 0.77 |
| H6 poultry | Constant | Lognormal | 3.6 (2.99-4.2) | 1993 (1990-1995) | 15716.04 | 1.56 |
| **H6 poultry** | **Constant** | **Strict** | **3.85 (3.38-4.31)** | **1994 (1993-1995)** | **15703.26** | **0.82** |

**Table 3: Estimates of substitution rate and time to the most recent common under different population size and clock priors for segment PB2.**

Mean and 95% Bayesian confidence intervals (between brackets) are reported for each gene. The best model for each data set according to the AICM is highlighted.

| Subtype | Population | Clock | Rate (95% HPD) | Age (95% HPD) | AICM | SE |
| --- | --- | --- | --- | --- | --- | --- |
| **H5N1** | **Skyride** | **Lognormal** | **3.82 (3.48-4.17)** | **2000 (2000-2001)** | **53015.499** | **14.929** |
| H5N1 | Skyride | Strict | 3.36 (3.12-3.6) | 1999 (1998-1999) | 53630.935 | 9.084 |
| H5N1 | Constant | Lognormal | 3.8 (3.45-4.17) | 1995 (1991-1999) | 53059.177 | 14.625 |
| H5N1 | Constant | Strict | 3.5 (3.24-3.76) | 1998 (1997-1999) | 53651.439 | 9.242 |
| H4 | Skyride | Lognormal | 2.31 (2.18-2.45) | 1965 (1963-1969) | 143818.51 | 26.33 |
| H4 | Skyride | Strict | 1.89 (1.81-1.97) | 1934 (1930-1937) | 143917.62 | 22.47 |
| **H4** | **Constant** | **Lognormal** | **2.14 (2-2.3)** | **1905 (1874-1934)** | **142982.89** | **25.75** |
| H4 | Constant | Strict | 1.87 (1.78-1.95) | 1908 (1900-1915) | 143742.37 | 16.65 |
| H6 | Skyride | Lognormal | 2.79 (2.61-2.96) | 1960 (1957-1962) | 131530.83 | 19.74 |
| H6 | Skyride | Strict | 2.15 (2.04-2.25) | 1929 (1925-1933) | 132588.78 | 12.43 |
| **H6** | **Constant** | **Lognormal** | **2.49 (2.31-2.65)** | **1909 (1876-1934)** | **131512.13** | **16.55** |
| H6 | Constant | Strict | 2.06 (1.96-2.16) | 1898 (1886-1908) | 132433.99 | 11.44 |
| H6 poultry | Skyride | Lognormal | 4.2 (3.2-5.23) | 1995 (1992-1998) | 14811.57 | 1.21 |
| H6 poultry | Skyride | Strict | 4 (3.45-4.58) | 1994 (1993-1996) | 14856.66 | 0.79 |
| **H6 poultry** | **Constant** | **Lognormal** | **3.99 (3.06-5.05)** | **1993 (1988-1998)** | **14811.44** | **1.22** |
| H6 poultry | Constant | Strict | 3.98 (3.39-4.53) | 1994 (1993-1996) | 14856.27 | 0.8 |
